# Isothermal Oxidation Behavior of Nickel Base Single Crystal DD6 Film-Cooling Blades at 1050 °C

**DOI:** 10.3390/ma18071498

**Published:** 2025-03-27

**Authors:** Chunyan Hu, Xinling Liu, Changkui Liu, Weikang Sun, Chunhu Tao

**Affiliations:** 1 AECC Beijing Institute of Aeronautical Materials, Beijing 100095, China; 2 Key Laboratory of Aeronautical Materials Testing and Evaluation, Aero Engine Corporation of China, Beijing 100095, China; 3 Beijing Key Laboratory of Aeronautical Materials Testing and Evaluation, Beijing 100095, China; 4 City University of Hong Kong Shenzhen Research Institute, Shenzhen 518057, China

**Keywords:** oxidation behavior, nickel base single crystal, superalloy, film cooling, growth stress

## Abstract

The isothermal oxidation behavior of single crystal DD6 film-cooling blades was investigated. The isothermal oxidation tests were conducted at 1050 °C, and the phase analysis was performed by XRD, while SEM (EDS) was employed to observe the material. In addition to experimental studies, a numerical simulation using three-dimensional finite element analysis based on Abaqus software (Version 6.13) was implemented to model the growth stress in specimens during the isothermal test. The obtained results showed that the average oxidation rate of specimens rose with increments in film hole spacing, up to a maximum value at a film hole spacing of 0.75 mm, and then fell, which could be interpreted with the concepts of the oxidation-affected zone and the growth stress. The results obtained from the numerical simulation of the growth stress agreed with the experimental results of the average oxidation rate. The oxide scale of film-cooling specimens mainly consisted of three layers, the NiO outer layer, the spinel sublayer containing cracks, and the non-continuous thin Al_2_O_3_ inner layer. The surface of the oxide scale commonly underwent spallation of the NiO outer layer, and the exposed sublayer could grow new NiO particles. The size of the NiO particles on the edge of the film holes was larger than those on the walls of the film holes. SEM images clearly showed that electro-hydraulic beam drilling on DD6 superalloy specimens could erode the γ phase in the γ/γ′ two-phase matrix, thereby inducing damages in regions near film holes.

## 1. Introduction

With the increase in the inlet temperature of gas turbine engines, oxidation resistance is becoming increasingly important and is a primary constraint limiting the widespread commercial application of the latest generation of SXs [1]. Therefore, oxidation behavior of SXs has been drawing more and more interests in recent years.

Previous work investigated the oxidation behavior of three prototype SXs for industrial gas turbine applications and indicated that the Al_2_O_3_-forming ability was stronger in the inter-dendritic regions than in the dendritic counterparts [2]. The isothermal oxidation tests also revealed the dependence of oxide microstructure on temperatures. The study on DD6 showed that the oxide structures consisted of an external NiO layer and an internal layer of Al_2_O_3_ at 1050 °C, while at 1100 °C, an external Al_2_O_3_ layer, an inter-layer of Cr_2_O_3_ and TaO_2_, and an internal layer of Al_2_O_3_ were formed [3]. Therefore, different types of SXs had distinct oxidation behavior in terms of oxidation kinetics, oxide composition, and distribution.

Several numerical studies have also been carried out to model the oxidation kinetics and the microstructure evolution process for different SXs. For example, in [4,5,6,7], Al_2_O_3_ growth was characterized with a parameter formulated from thermodynamic and kinetic principles, and a model for substrate response was employed to calculate the γ′ precipitate evolution. The numerical results obtained by these authors were proved to have quite good consistency with the experimental data. The oxide morphology change of Ni-Al and Ni-Cr-Al alloys was successfully modelled with simplifying assumptions in previous work [8].

Factors affecting the oxidation behavior of SXs, such as element addition, grain size, water vapor involvement, temperature, loading condition, and so on, were also examined previously [9,10,11,12,13,14,15]. Grain refinement can boost the diffusivity of Cr in alloy N as suggested in previous work [10]. Previous studies found that the primary mechanism of oxidation-induced damage included the initiation of external oxide structures and internal defects (micro-cracks and oxide particle inclusions), which could be aggravated by higher temperatures [12]. In applications, thin-walled turbine blades with film-cooling holes are commonly used. The existence of film holes boosts the cooling efficiency to a great extent [13], whereas it also brings two detrimental effects simultaneously: one is the decrease in mechanical properties [14], and the other is the oxidation-caused geometrical disintegration [15].

Previous studies commonly use smooth specimens (either thin plates or cylindrical bars) to model the oxidation behavior of SX turbine blades. As the geometrical difference caused by film holes can have notable effects on stress states and finally the creep rupture performance of SX blades [16,17], it may also have influence on the oxidation behavior of SX blades; however, very limited research has been conducted to investigate this important problem. In this paper, we present the influence of film hole spacing on the oxidation kinetics of thin-walled DD6 specimens and the oxide morphology observation results.

## 2. Materials and Methods

### 2.1. Materials

The material utilized in this research was DD6, a second-generation single crystal superalloy developed by the Beijing Institute of Aeronautical Materials, and had many advantages such as high strength at elevated temperature, excellent comprehensive performance, and good microstructure stability. Its nominal composition is shown in Table 1 [16].

### 2.2. Isothermal Oxidation Test

The film hole spacing of the samples was designed according to the real hole spacing of the gas film holes in turbine blades.

**Figure 1:**
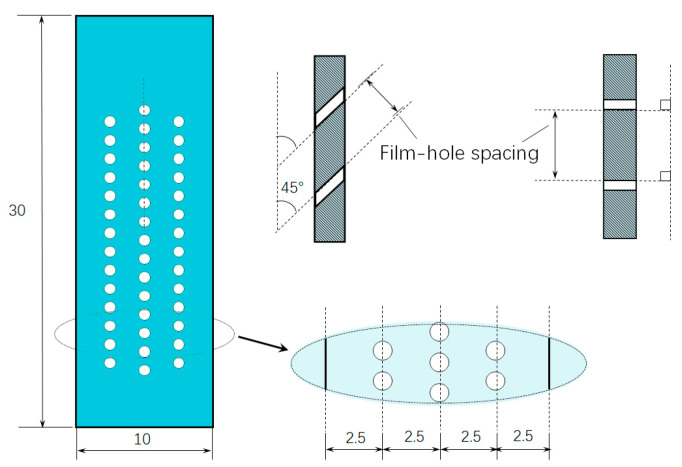
Diagram of the specimen with inclining film holes (dimension: mm).

Plate specimens with 3 rows of film holes were prepared for isothermal oxidation tests. As shown in Figure 1, each type of specimen was rectangular with an even thickness of 1.5 mm and with film holes of 0.4 mm in diameter, using an electro-stream machining (ESM) technique. The design strategies of the specimens were as follows: (1) a constant number (43) of film holes were machined for all specimens, (2) the specimens were axisymmetric to the longitudinal axis, and (3) the central axis of the film hole was inclined at either at 45° or 90° to the longitudinal axis.

Two types of specimens with varying film hole spacing were manufactured and numbered as displayed in Table 2. Each model had three repetitive specimens, and the experiment results were assessed with the average value of the three repetitive specimens.

Isothermal oxidation tests of each specimen were conducted with a furnace. Specimens were kept at 1050 °C in dry air for 900 h, during which specimens were taken out for morphology observation and mass measurement every 50 h.

In order to be closer to the actual service conditions of turbine blades and to better observe the variation trend of oxidation weight gain under different hole spacings, the heating time was extended to 900 h.

### 2.3. Phase Analysis and Morphology Observation

The X-ray diffraction study for phase analysis was carried out using a Bruker AXS D8 Advance diffractometer with Cu Kα-radiation(SC-XRD, Karlsruhe, Germany). The spectrum peaks were matched with JCDPS data, and the main oxides and structures corresponding to DD6 specimens were determined.

A scanning electron microscope was utilized to investigate the oxide microstructures and morphology (SEM, Zeiss Gemini SEM500, Jena, Germany). In addition, a transmission electron microscope was also employed to display the microstructure in the vicinity of the film holes.

### 2.4. Numerical Simulation

**Figure 2:**
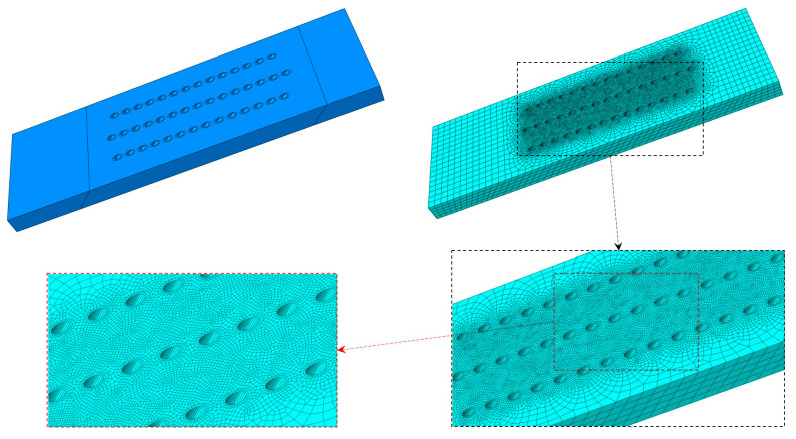
Geometrical and FE model of a representative specimen with slanted film holes.

Finite element analysis (FEA) with Abaqus software (Version 6.13) was conducted to simulate the growth stress in specimens during the isothermal test. The geometrical and FEA model of a representative specimen is shown in Figure 2.

The thermal expansion scenario was used to model the oxide growth and squeezing as they exhibited rather similar mechanical behavior. The geometrical parameters for FEA models were identical to the specimens used in isothermal test. Partitions were conducted, and thin walls around the film holes with 0.1 mm thickness were taken to model the oxide layers. Two types of materials were utilized to model the matrix and oxide layer, which had the same elastic properties but different thermal expansion coefficients. Two categories of mechanical properties were chosen in the numerical analysis: one was the isotropic material model (simplified model), and the other was the orthotropic model with [100] direction assigned in accordance to the longitudinal axis of the specimens. The material properties are shown in Table 3.

## 3. Experimental Results

### 3.1. Oxidation Kinetics

Average oxidation rate *K* was determined according to reference [18],

$$K=\frac{m_{2}-m_{1}}{850\cdot A}$$

where $m_{1}$ and $m_{2}$ are the total mass of the specimen and the container at 50 h and 900 h, respectively, and *A* is the oxidized area of the specimen.

**Figure 3:**
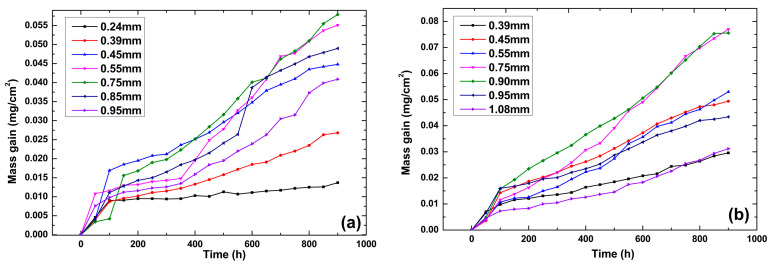
Oxidation kinetics curves of different specimens with varying film hole spacing: (**a**) specimens with slanted film holes and (**b**) specimens with straight film holes.

**Figure 4:**
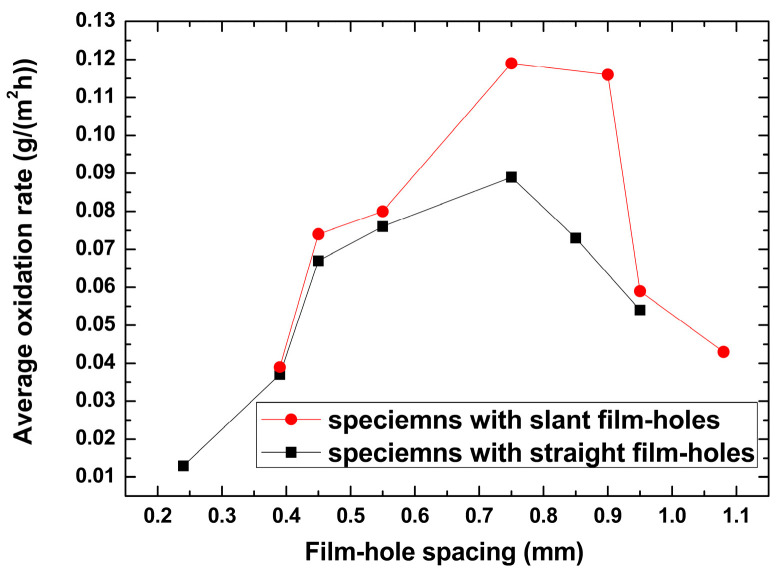
Average oxidation rates of different specimens with varying film hole spacing.

The oxidation kinetics curves and average oxidation rate results of different specimens are shown in Figure 3 and Figure 4. The results showed that the average oxidation rate for specimens with the film hole spacing of 0.75 mm was faster than those of other specimens for both film hole drilling cases. The oxidation sped up in the beginning, which was the initiation period of the oxide scale. After 100~150 h, the oxidation process slowed down, which was related to the oxide scale growth period. The average oxidation rate increased with the film hole spacing augmented from the minimum to 0.75 mm and then showed an opposite trend (Figure 4). The average oxidation rate for specimens with slanted film holes was greater than those observed for specimens with straight film holes, and this difference became more obvious when the film hole spacing ranged from 0.7 mm to 0.9 mm.

### 3.2. Phase Analysis

**Figure 5:**
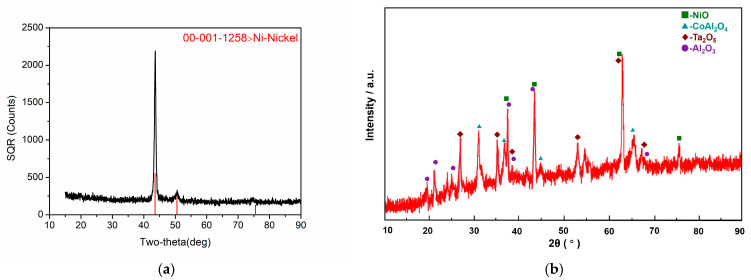
Phase analysis of oxide layers by XRD: (**a**) before the oxidation test and (**b**) after the oxidation test.

Figure 5 presents the XRD analysis results of the DD6 oxide layers before and after 900 h oxidation at 1050 °C. It could be seen that the oxidation products formed on the substrate were mainly NiO, Al_2_O_3_, a small amount of Ta_2_O_5_, and spinels like CoAl_2_O_4_. The results of XRD analysis were obviously different from those of the samples before oxidation.

### 3.3. Morphologies and Microstructures

**Figure 6:**
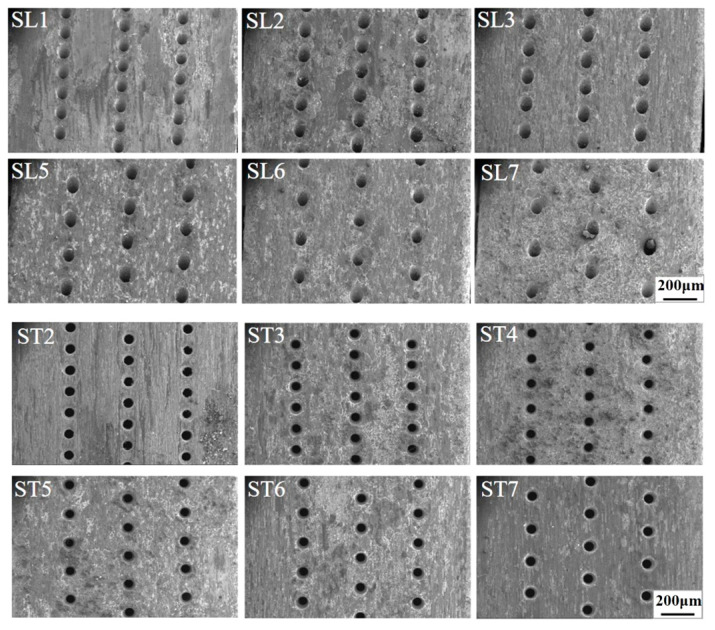
Macroscopic morphology in local regions of the representative specimens.

Figure 6 displays the macroscopic morphology of the specimens after the 900 h isothermal oxidation test. Spallation of oxide layers can be seen even more clearly in Figure 6, such as the middle region in SL7 and the rough surface in ST4.

**Figure 7:**
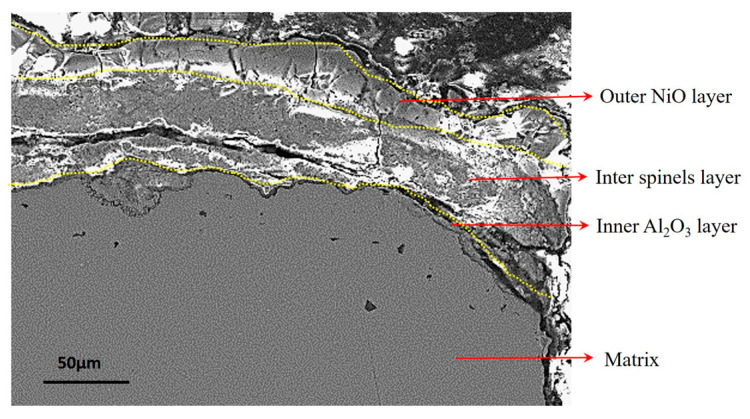
Cross-sectional morphology and microstructures of the specimens.

Combined with the cross-sectional morphology observation of the oxide scale and the EDX analysis, the oxide scale was observed to be composed of three layers (Figure 7): an outer NiO layer with a small amount of CoO; an inter-layer (or sublayer), mainly consisting of spinels like NiCr_2_O_4_ and NiAl_2_O_4_; and an inner Al_2_O_3_ layer. This result was consistent with previous experimental results. However, the Al_2_O_3_ layer was very thin and discontinuous. Furthermore, multiple cracks were also observed in the inter-spinel layer.

**Figure 8:**
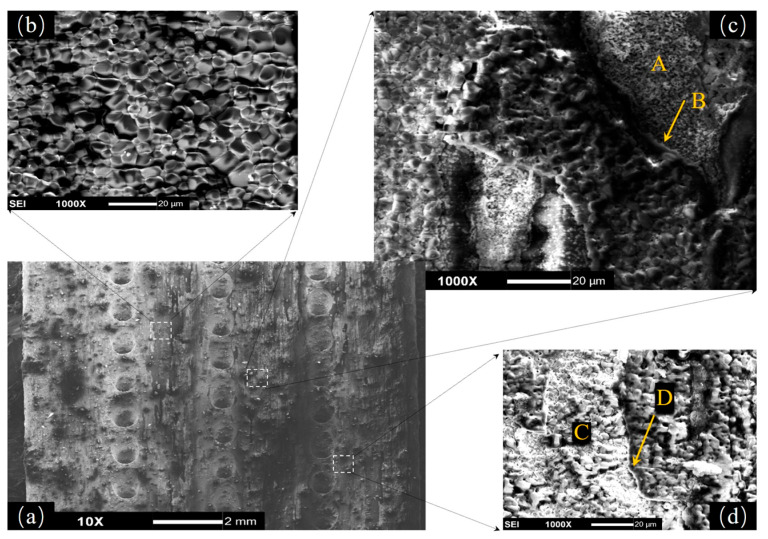
Surface morphology and microstructures of DD6 isothermal oxidation specimens with film holes: (**a**) macroscopic pattern and (**b**–**d**) microscopic pattern.

Figure 8 presents SEM images of the macroscopic and microscopic morphology of DD6 specimens after the 900 h isothermal test at 1050 °C. In some regions, NiO particles lay against each other, forming perfect continuous NiO outer layer (Figure 8b). However, in most areas, spallation of oxide layers was a very common phenomenon. For example, in Figure 8c, a clear spallation boundary (marked with B) could be observed, and without shelter, a fresh sublayer consisting mainly of smaller sized spinels like NiAl_2_O_4_ was exposed in air (marked with A). In Figure 8d, the boundary of spallation still could be identified (marked with D), but in this case, the sublayer was mixed with both spinels and newborn NiO particles (marked with C).

**Figure 9:**
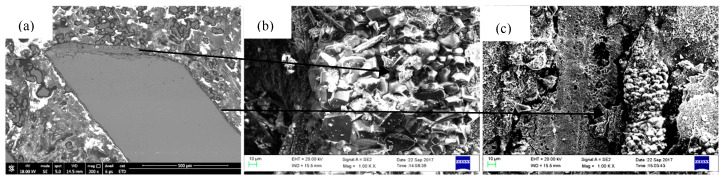
Surface morphology and microstructures of DD6 oxide layer in the vicinity of film holes: (**a**) macroscopic pattern and (**b**,**c**) microscopic pattern.

Figure 9a displays the cross-sectional morphology of DD6 specimens in regions near the film holes. Compared with those on the wall of the film hole, the oxide scale at the edges of the film holes was thicker, and the size of the oxide particles was larger (Figure 9b,c).

### 3.4. Thin-Wall Effect of Oxidative Damage on the Film Holes

**Figure 10:**
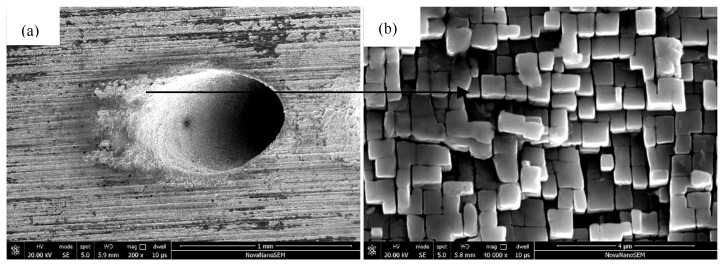
Electrolytic corrosion at the edges of holes: (**a**) macroscopic pattern and (**b**) microscopic pattern.

Figure 10a shows the macroscopic appearance of electrolytic corrosion on the inner walls of the film holes after electro-hydraulic beam (EHB) drilling on DD6 superalloy. As revealed in Figure 10b, the electrolysis corrosion layer infiltrated into the substrate material. It could be observed that some of the γ phase was corroded, and the γ′ phase became prominent. EHB drilling will inevitably cause corrosion damage at the edges of holes of the DD6 superalloy and deteriorate the properties of the protective oxide layers. Nickel matrix reduction on the edges of film holes can be an important reason for the larger size of NiO particles in this region since a lower content of nickel element means less competition for oxygen, and each NiO particle can grow with relatively abundant oxygen.

**Figure 11:**
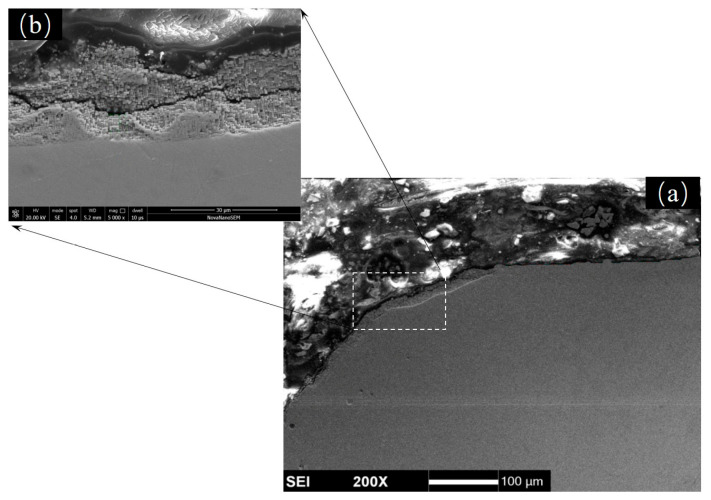
SEM images of the electrolytic corrosion layer at the edges of holes: (**a**) macroscopic pattern and (**b**) microscopic pattern.

Figure 11 shows the cross-sectional morphology of the electrolytic corrosion at the edges of holes. Fractures in the corrosion layers can be observed clearly (Figure 11b), which may lead to the cracks in the inter-layer of the oxide scale. Because the protective Al_2_O_3_ layer was the innermost oxide layer, which just formed on the matrix of alloys, the existence of these fracture may retard the formation of a continuous Al_2_O_3_ layer and, to some extent, promote the oxidation rate of DD6 specimens.

### 3.5. Numerical Results

**Figure 12:**
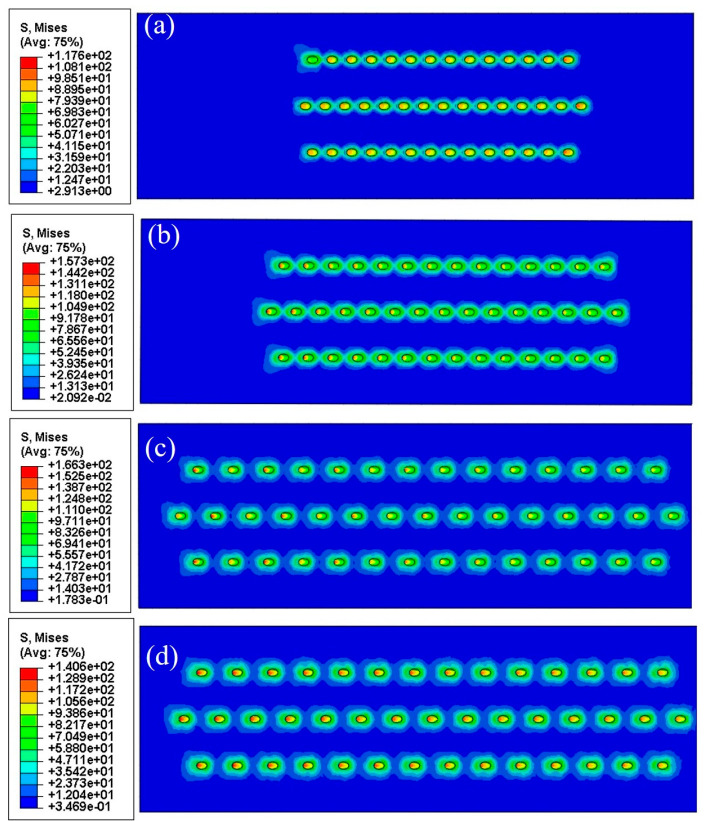
Growth stress distributions of representative specimens with slanted cooling holes and film hole spacings of (**a**) 0.35 mm, (**b**) 0.55 mm, (**c**) 0.75 mm, and (**d**) 0.95 mm.

**Figure 13:**
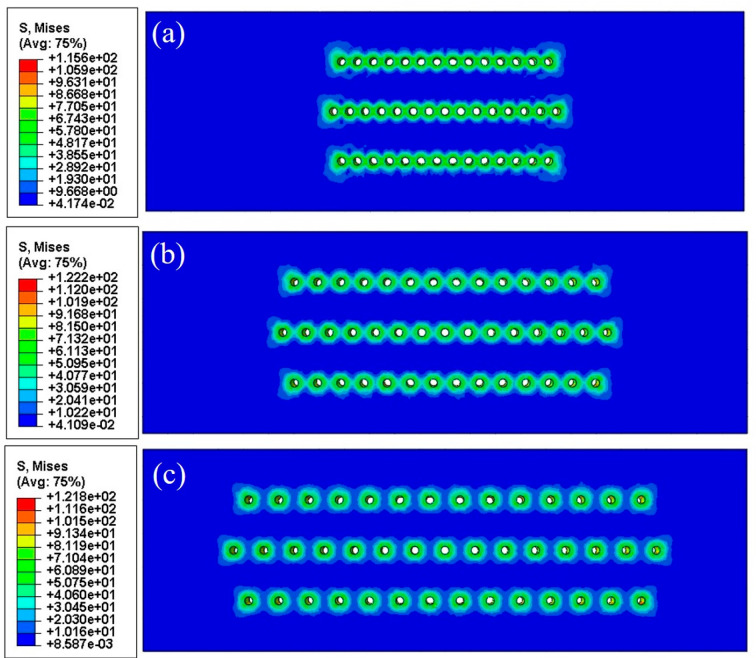
Growth stress distributions of representative specimens with straight cooling holes and film hole spacings of (**a**) 0.45 mm, (**b**) 0.75 mm, and (**c**) 1.1 mm.

**Figure 14:**
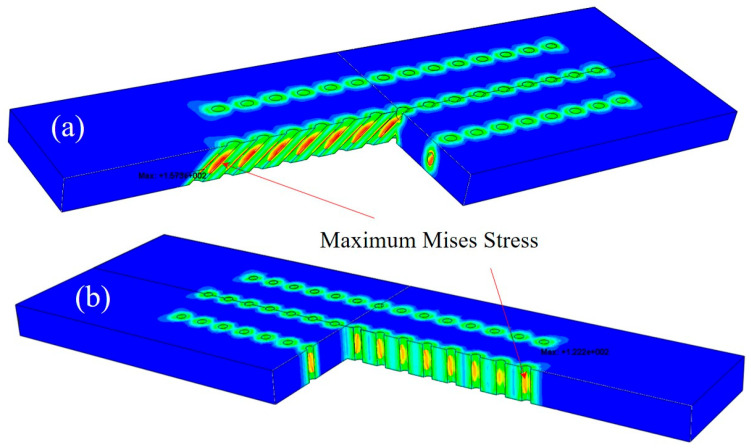
Three-dimensional growth stress distributions of representative specimens with (**a**) slanted film holes and (**b**) straight film holes.

Numerical results are shown in Table 4 and Figure 12 and Figure 13. The number format in the legend of stress contours is “exponent” (e.g., 1e+02 is 1 × 10^2^).The growth stress distributions were quite similar for both types of specimens with slanted and straight film holes. With the increment of film hole spacing, high-stress regions gradually separated from the connected mode, and each film hole would withstand the increasing stress concentration effect independently. It can be observed from Figure 14 that the maximum Mises stresses were located in the middle area on the inner walls of the film holes, which lay on one side of the specimens.

As can be seen in Table 4, the maximum Mises stresses for the orthotropic material model were smaller than the figures for the isotropic one, which was probably caused by the relatively low value of the Young’ modulus for the orthotropic model. Regardless of this difference, with the increase in the film hole spacing, both models showed an up-and-down changing trend of maximum Mises stress. When the film hole region kept a distance from the two longitudinal free ends of specimens, the nearer two film holes were located, the smaller the stress concentration effect would become. This was because the neighboring film holes could share some of the concentrated stress as presented in Figure 14a,b. But beyond the certain distance from the free ends, less constraint of deformation from the free boundary surfaces lessened the stress concentration effect.

## 4. Discussion

### 4.1. Oxidation Kinetics of Specimens with Different Film Hole Spacings

Oxide layer growth will produce stress at the interface between the alloy matrix and the oxide film, known as growth stress. The growth stress at an air film hole not only affects the stress state around that hole but also influences the stress states of adjacent holes, a phenomenon referred to as the stress coupling superposition effect. During the growth process, the oxide film will fall off due to factors such as mismatch of thermal expansion coefficient and thermal stress. After the oxide film falls off, it lacks the ability to block the O element, and the oxidation degree will be further aggravated.

Transient oxidation rates for different specimens showed distinct features throughout the isothermal test process. Specimens with slanted film holes and film hole spacings of 0.24 mm and 0.39 mm displayed relatively high stabilities in oxidation rate, while those for other sets of specimens showed varying oxidation rates in different testing periods. This instability in the transient oxidation rate may result from the stimulating effect due to spallation of the oxide layers. The curve of the average oxidation rate vs. the film hole spacing rose from 0.24 mm, peaked at 0.75 mm, and fell after the extremal point. This non-monotonic pattern could be attributed to two dominant factors, which were the size of the oxidation-affected zone and the growth stress in the oxide layers.

### 4.2. Oxide Layers and Oxidation Affected Zone

References [19,20] suggested that the thickness of the γ′ reduced layer increased with the increment of the initial specimen thicknesses until it achieved the maximum value (about 0.4 mm). Oxidation is a process of ion diffusion, and the size of the γ′ reducing layer could represent the size of the oxidation-affected zone (OAZ) to some extent. With the increment of film hole spacing, OAZ expanded to the critical value, beyond which the size of the OAZ did not change any more. This could account for the rising part of the oxidation rate curve.

### 4.3. Growth Stress in Oxide Layers

References [21,22] pointed out that oxide growing stress existed due to the oxide particles growing and stacking on the surface of the matrix and the mismatching of the oxide layer with the matrix layer of superalloys. Through the mechanics simulation described in Section 2.4, it could be found that with the increment film hole spacing, the growing stress near the film holes decreased. Residual stress, namely, the growing stress, could accelerate the spallation of oxide layers, which promoted the oxidation rate.

The maximum von Mises stresses for the two material models both peaked at the film hole spacing of 0.75 mm, which were consistent with the oxidation rate results. The maximum von Mises stress with a 0.75 mm film hole spacing for the slanted film hole case was greater than the figure for the straight film hole counterpart, which again coincided with the average oxidation results. This meant that the oxidation rate results could be interpreted with the concept of growth stress. Generally, the geometrical difference can cause distinct growth stress levels, which lead to the corresponding oxide layer spallation rate distinction and eventually the average oxidation rate difference.

## 5. Conclusions

In this paper, we examined the oxidation behavior for the DD6 superalloy with film holes using experimental and numerical approaches. An isothermal oxidation test was conducted at 1050 °C for 900 h, and oxidation kinetics characterization, phase analysis, microstructure observation, and numerical simulation were conducted. Major conclusions are summarized below.

With the increment of film hole spacing, the average oxidation rate of specimens rose to the maximum value at a film hole spacing of 0.75 mm and then fell. This phenomenon could be attributed to the interaction between effects imposed by the size of oxidation-affected zone and the growth stress in oxide layers. The numerical results of the growth stress coincided with the experimental results of the average oxidation rate.

The oxide scale of film-cooling specimens consisted of three layers: the NiO outer layer, the spinel inter-layer, and the inner Al_2_O_3_ layer. The surface of the oxide scale commonly underwent spallation of the NiO outer layer, and the exposed sublayer could grow new NiO particles in spinels. The NiO particles at the edges of film holes were larger in size than those grown on the walls of the film holes.

SEM images clearly showed that electro-hydraulic beam drilling on DD6 superalloy specimens can erode the γ phase in the γ/γ′ two-phase matrix and cause damages in the surface of regions near film holes. This can account for the fractures observed in the inter-layer of the oxide scales.

## Figures and Tables

**Table 1:** Nominal compositions of DD6 superalloy (mass fraction %).

Co	W	Ta	Al	Cr	Re	Mo	Ni
9	8	7.5	5.6	4.3	2	2	Balance

**Table 2:** Information table of the isothermal oxidation specimens.

Specimen No.	Drilling Angles to the Longitudinal Axis (°)	Film Hole Spacing (mm)	Specimen No.	Drilling Angles to the Longitudinal Axis (°)	Film Hole Spacing (mm)
SL1	45	0.24	ST1	90	0.39
SL2	45	0.39	ST2	90	0.45
SL3	45	0.45	ST3	90	0.55
SL4	45	0.55	ST4	90	0.75
SL5	45	0.75	ST5	90	0.9
SL6	45	0.85	ST6	90	0.95
SL7	45	0.95	ST7	90	1.08

**Table 3:** Material properties used in FEA simulation.

Material Model		Young’s Modulus (GPa)	Poisson’s Ratio	Thermal Expansion Coefficient (10^−6^)
Matrix	Isotropic	200.0	0.30	1.0
	Orthotropic ([001])	80.5	0.39	1.0
Oxide layer	Isotropic	200.0	0.30	5.0
	Orthotropic ([001])	80.5	0.39	5.0

**Table 4:** Numerical results of growth stress simulation of the isothermal specimens.

Specimens	Film Hole Spacing (mm)	Maximum Mises Stress of FEM Models (MPa)	
		With Isotropic Materials	With Orthotropic Materials
With slanted film holes	0.35	135.9	117.6
	0.55	173.5	157.3
	0.75	187.3	166.3
	0.95	154.6	140.6
With straight film holes	0.4	147.7	115.6
	0.75	153.6	122.2
	1.1	151.7	121.8

## Data Availability

Data are contained within the article.
